# Safety and feasibility of radiofrequency ablation using bipolar electrodes for aldosterone-producing adenoma: a multicentric prospective clinical study

**DOI:** 10.1038/s41598-022-18136-5

**Published:** 2022-08-18

**Authors:** Sota Oguro, Ryo Morimoto, Kazumasa Seiji, Hideki Ota, Tomo Kinoshita, Masahiro Kawabata, Yoshikiyo Ono, Kei Omata, Yuta Tezuka, Fumitoshi Satoh, Sadayoshi Ito, Nobukazu Moriya, Seishi Matsui, Tetsuo Nishikawa, Masao Omura, Kazuki Nakai, Seishi Nakatsuka, Isao Kurihara, Kazutoshi Miyashita, Wataru Koda, Tetsuya Minami, Yoshiyu Takeda, Mitsuhiro Kometani, Yutaka Oki, Toshihiro Oishi, Takasuke Ushio, Satoshi Goshima, Kei Takase

**Affiliations:** 1 Diagnostic Radiology, Tohoku University School of Medicine, Tohoku University Hospital, 1-1 Seiryocho, Aoba-ku, Sendai, Miyagi 980-8574 Japan; 2 Nephrology, Endocrinology and Vascular Medicine, Department of Medicine, Tohoku University Hospital, Sendai, Miyagi Japan; 3 Department of Radiology, South Miyagi Medical Center, Ōgawara, Miyagi Japan; 4 Division of Clinical Hypertension, Endocrinology & Metabolism, Tohoku University Graduate School of Medicine, Sendai, Miyagi Japan; 5 Department of Radiology, Yokohama Rosai Hospital, Yokohama, Kanagawa Japan; 6 Endocrinology and Diabetes Center, Yokohama Rosai Hospital, Yokohama, Kanagawa Japan; 7 Department of Diagnostic Radiology, Keio University School of Medicine, Tokyo, Japan; 8 Department of Medical Education, National Defense Medical College, Tokorozawa, Saitama Japan; 9 Division of Endocrinology, Metabolism and Nephrology, Department of Internal Medicine, Keio University School of Medicine, Tokyo, Japan; 10 Department of Radiology, Kanazawa University Graduate School of Medical Science, Kanazawa, Ishikawa Japan; 11 Department of Health Promotion and Medicine of the Future, Graduate School of Medical Science, Kanazawa University, Kanazawa, Ishikawa Japan; 12 Department of Internal Medicine, Graduate School of Medical Science, Kanazawa University, Kanazawa, Ishikawa Japan; 13 Department of Diabetes/Endocrinology, Hamamatsu University School of Medicine, Shizuoka, Japan; 14 Department of Radiology, Hamamatsu University School of Medicine, Shizuoka, Japan; 15 Department of Diabetes/Endocrinology, Hamamatsu-Kita Hospital, Shizuoka, Japan

**Keywords:** Endocrinology, Urology

## Abstract

Evaluation of feasibility and safety of percutaneous radiofrequency ablation using bipolar radiofrequency devices in a prospective multicenter cohort of patients with benign aldosterone-producing adenoma. A total of five institutions participated. CT-guided percutaneous RFA was performed for patients diagnosed as APA. The safety of the procedure was evaluated using the Common Terminology Criteria for Adverse Events. During the 84-day follow-up period, serial changes in plasma aldosterone concentration and plasma renin activity were measured. The percentage of patients with normalized hormonal activity after the procedure, was calculated with 95% confidence intervals. Forty patients were enrolled, and two patients were excluded for cerebral hemorrhage and no safe puncture root. In another patients, RFA was tried, but an intraprocedural intercostal arterial injury occurred. Consequently, RFA was completed in thirty-seven patients (20 men, 17 women; mean age, 50.4 ± 10.0 year). The tumor size was 14.8 ± 3.8 mm. The treatment success rate of the ablation was 94.6% (35/37), and a 2nd session was performed in 2.7% (1/37) patients. Grade 4 adverse events were observed in 4 out of 38 sessions (10.5%). The normalization of plasma aldosterone concentration or aldosterone-renin ratio was 86.5% (72.0–94.1: 95% confidence interval) on day 84. Percutaneous CT-guided RFA for APA using a bipolar radiofrequency system was safe and feasible with clinical success rate of 86.5% on day 84.

## Introduction

Aldosterone-producing adenoma (APA) is a benign adrenal tumor that causes primary aldosteronism (PA) and secondary hypertension. Patients with PA are reported to be more likely to have cardiovascular complications at diagnosis^1–3^. Laparoscopic adrenalectomy has been recommended for unilateral APA, except in patients who are not indicated for or are reluctant to undergo surgery^3,4^. In patients with unilateral APA, surgical treatment provides a better quality of life and is more cost-effective, as it reduces the use of antihypertensive drugs and has long-term cost benefits^5–9^. Although laparoscopic adrenalectomy is regarded as less invasive than open surgery, the potential risks and invasiveness of performing procedures under general anesthesia should be considered. Alternatively, less-invasive radiofrequency ablation (RFA) has been proposed for patients who are reluctant to undergo surgery or are not indicated for surgery^10–13^.

Liu et al*.* reported that computed tomography (CT)-guided percutaneous RFA is a safe technique for treating small APAs^14^. They performed CT-guided RFA for 24 APA patients using monopolar RF devices with water running through the probe tip by 12-min cycle of ablation. The procedure was a clinical success in 95.8% patients. However, the monopolar device has several limitations, such as a long ablation time, small ablation volume, and risks of skin burns due to ground pad heating. Recently, bipolar RF devices have been developed. They are being used for liver and renal tumors and have been reported to provide shorter ablation time and larger ablation volume than monopolar RF devices^15,16^. In addition, when multiple bipolar RF devices were placed, the target lesion was ablated in several direction between each electrode using a computer-controlled switching^16^. The feasibility of percutaneous RFA using bipolar RF devices for APA has not been previously reported. The purpose of this study was to evaluate the feasibility and safety of percutaneous RFA using bipolar RF devices in a prospective multicenter cohort of patients with benign APAs.

## Methods

This prospective trial was approved by the Independent Ethics Committee of Tohoku University School of Medicine (C-01-2). A total of five institutions participated in the single-group, quasi-experimental, non-controlled, non-blinded, multicenter study between January 2015 and September 2016. Investigator-initiated clinical trial was approved by the Ministry of Health, Labor and Welfare. This trial was registered in the University Hospital Medical Information Network Clinical Trial Registry (UMIN-CTR) which is one of Japan Primary Registries Network (JPRN) approved by WHO, and the trial registration data are available on UMIN000015865, 08/12/2014. All methods were performed in accordance with the Declaration of Helsinki.

### Study population and diagnosis of APA

#### Inclusion criteria

All patients were diagnosed with unilateral APA by conventional adrenal vein sampling (AVS) by administration of 0.25 mg cosyntropin^17,18^. The adrenal venous blood aldosterone/cortisol (A/C) ratio after ACTH stimulation is calculated bilaterally, and if the lateralized ratio = (adrenal vein A/C ratio on the high-value side) ÷ (adrenal vein A/C ratio on the low-value side) is ≥ 2.6, a unilateral lesion is considered to be present on the high value side^17^. Location of the adrenal adenoma was examined on dynamic CT in all cases. MRI was not mandatory in this study. Inclusion criteria were as follows: (a) Patients above 20 years of age who provided informed consent; (b) those diagnosed with unilateral single APA by AVS within 3 years; (c) those without any organs at dangerous locations, i.e., small and large bowel, large vessels, and pancreas/kidney placed on the puncture route; (d) those with less than 5 mm of intervening adipose tissue between the APA and pancreas/small intestine; (e) well defined tumors with the size of the lesion more than 5 mm and less than 25 mm on CT images; (f) white blood cell count > 3000/mm^3^, hemoglobin level was > 8.0 g/dL, and estimated glomerular filtration rate > 45 mL/min./1.73m^2^; (g) Performance status < 2.

#### Exclusion criteria

Exclusion criteria were as follows: (a) The contralateral adrenal glands showing severe hypofunction or those after contralateral adrenalectomy; (b) those with the possibility of a malignant ipsilateral adrenal tumor; (c) those with prothrombin time and international normalized ratio > 2.0; (d) those with signs or symptoms of infection; (e) those with a cerebral hemorrhage, Cushing syndrome, paraganglioma, pregnancy, or a history of allergy to iodinated contrast media; (f) those to whom only a trans-pulmonary puncture route allows for accessing the target lesion in the RFA procedure.

#### RFA procedure

CT-guided percutaneous RFA of APA was performed using a multipolar RFA device (CELON POWER System, Olympus, Japan). The qualification of the operator was a physician who is qualified as an interventional radiology board certified specialist, who has experience in at least 50 cases of CT-guided puncture, and who is able to perform emergency embolization for postoperative hemorrhage. All the patients were placed in ipsilateral side down position to provide a safer route for puncturing the adrenal gland, and the optimal access route for RFA needle insertion was planned. Respiratory and blood pressure control management was provided by anesthesiologists. Oxygenation was maintained adequately by a more than five-liter oxygen mask. No patients were intubated. For analgesia and sedation, an appropriate amount of fentanyl and dexmedetomidine was injected intravenously. The antihypertensive drug e.g. alpha blocker, beta blocker, and calcium channel blocker was administered intravenously during the procedure.

**Figure 1:**
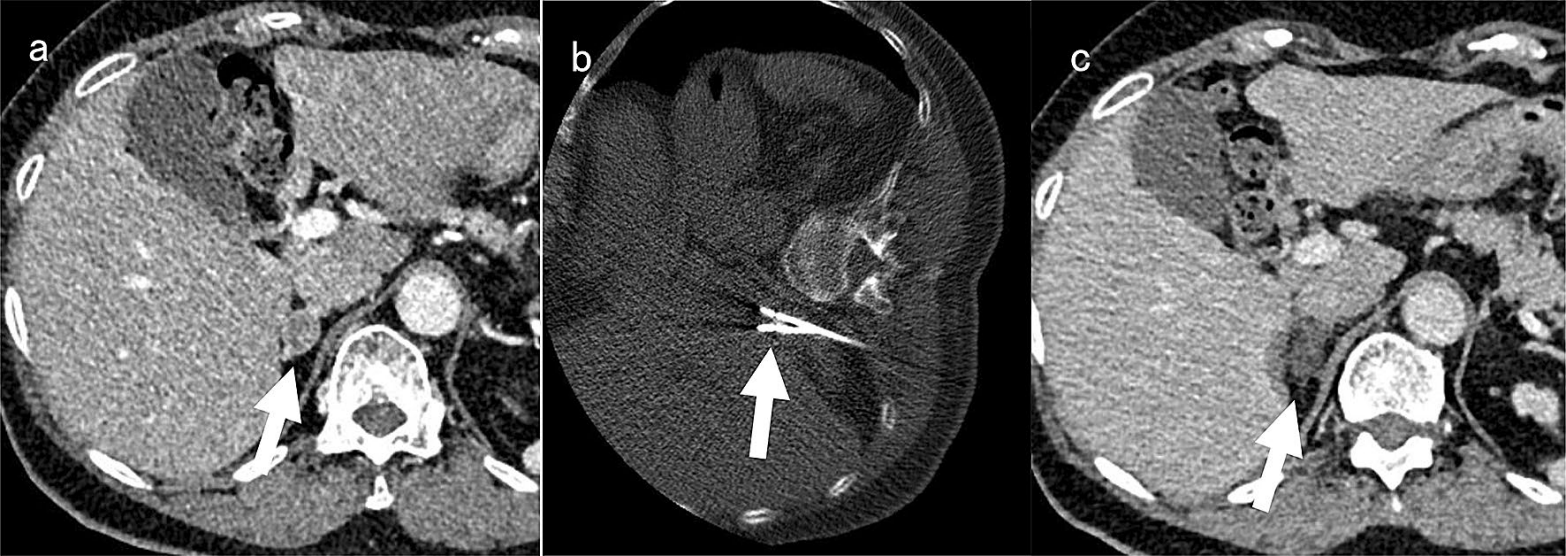
An example of CT-guided radiofrequency ablation for right adrenal adenoma. (**a**): Pre-procedural dynamic CT showed14 mm well-circumscribed right adrenal adenoma (arrow). (**b**): Two electrodes were inserted into the tumor by CT-guidance in the right lateral decubitus position, then radiofrequency ablation was performed (arrow). (**c**): The adrenal adenoma did not show contrast enhancement on post-procedural dynamic CT (arrow). A small part of a liver tissue surrounding the right adrenal gland showed low attenuation because of radiofrequency ablation.

After local anesthetic infiltration with 5–10 mL of plain lignocaine 1%, a 1.8 mm thick bipolar RFA device (CELON ProSurge applicator, Olympus, Japan) was inserted into the adrenal adenoma under CT guidance (Fig. 1a,b). One electrode was inserted into the lesion less than 10 mm in diameter, and the output power and total amount of energy were set as 20 W and 4 kJ, respectively. Two electrodes were inserted into the lesion from 10 to 25 mm in diameter, and the output power and total amount of energy were set as 40 W and 6 kJ, respectively, for each ablation. The electrode was circulated internally by chilled water. Immediately after the ablation, contrast-enhanced CT was performed to evaluate the extent of tumor ablation by injecting 50 ml of 300 mgI/ml Iohexol (Fig. 1c). Incomplete ablation was defined as a residual tumor enhancement on dynamic contrast-enhanced CT. In cases of incomplete ablation, additional RFA was performed consecutively.

Following the 1st RFA procedure, dynamic contrast-enhanced CT was performed after 7 days. In case of incomplete ablation, the 2nd RFA session was performed within 84 days after the 1st session.

CT fluoroscopy was used under the following scan protocols: tube voltage, 120 kVp; tube current, 20 mA; slice thickness 4 mm; number of slices 3 per scan, and matrix size, 512 × 512. Helical CT scan to confirm the locations of the needles just before the ablation was performed as follows: tube voltage, 120 kVp; tube current, real exposure control system (SD 28:30 ~ 70 mA); rotation speed, 0.5 s; Helical Pitch, 15 and matrix size, 512 × 512.

#### Evaluation of RFA procedure

The number of ablations, ablation time per cycle, overall output time per session, deployed energy, and total procedure time from the administration of first local anesthesia till departure from the operating room were recorded. To assess safety, systolic non-invasive blood pressure (NIBP) during the procedure was monitored every 5–10 min and was continuously measured during the RFA. Hypertensive crisis is defined by the Joint National Committee on Detection, Evaluation, and Treatment of High Blood Pressure version 7 as an increase of systolic blood pressure greater than 180 mm Hg or an increase in diastolic pressure greater than 120 mm Hg^19–21^. The frequency and severity of adverse events were assessed based on the Common Terminology Criteria for Adverse Events version 4.0^22^. The dynamic contrast-enhanced CT performed 7 days after the final RFA procedure showed the percentage of ablated volume in the total tumor volume, which was analyzed independently by a radiologist who was an image interpreter at a third-party organization. Technical success was defined as 100% ablated tumor volume; otherwise, the procedure was defined as a technical failure.

#### Evaluation of clinical outcome and endpoints

Plasma aldosterone concentration (ng/dL) and plasma renin activity (ng/mL/h) were measured at 3, 7, 28, and 84 days after the procedure. Normalization of hormone activity was defined as either plasma aldosterone concentration < 15 ng/dL or aldosterone-renin ratio (ARR) < 30. The primary endpoint was defined as the normalization of hormone activity on day 84. The number of antihypertensive drugs required for the management of hypertension was also recorded.

#### Sample size estimation

A single-center exploratory study including 8 patients with unilateral primary aldosteronism had been performed in the corresponding author’s institution prior to this multi-center study. Inclusion and exclusion criteria for the exploratory study had been identical. Bipolar RFA to the unilateral tumor had achieved biochemical normalization in all patients (100%) with no significant complication on day 84. A previous study by Novitsky et al. demonstrated that unilateral adrenalectomy for APA resulted in eradication of hypokalemia and resolution or significant improvement in hypertension in 80% patients at long-term follow-up^23^. According to these data, our multidisciplinary team including endocrinologists, interventional radiologists and urological surgeons reached consensus as follows: the less invasive RFA procedure would be acceptable as a treatment option of unilateral APA if the expected success rate of 95% with the lower limit of 80%. Estimated sample size was calculated as 35 under one-sided alpha level of 0.025 and beta level of 0.2. Considering a few dropped-out cases, we determined to enroll a total of 40 patients.

#### Statistical analysis

Frequencies and percentages for categorical variables and mean ± standard deviation (SD) for continuous variables were generated. The number and types of antihypertensive drugs before and after RFA treatment was evaluated using the Wilcoxon signed-rank test. Success rate of clinical outcome, ie, the percentage of patients with normalized hormonal activity after the RFA procedure, was calculated with 95% confidence intervals. Statistical significance was set at *p* < 0.05. Analysis was conducted using SAS Release 9.2. and SPSS version 25.

## Results

A total of 42 patients consented and 40 patients were enrolled for this study. The adrenal adenoma was well visualized on dynamic CT in all cases. Refractory hypokalemia was observed in 16 cases. Out of the 40 patients, one patient had a cerebral hemorrhage 15 days before the scheduled procedure. Another patient was declared unfit for the puncture on the day of the RFA procedure because of the surrounding lung seen on the planning CT images. Performance status was 0 in all patients.

### Safety and feasibility

**Figure 2:**
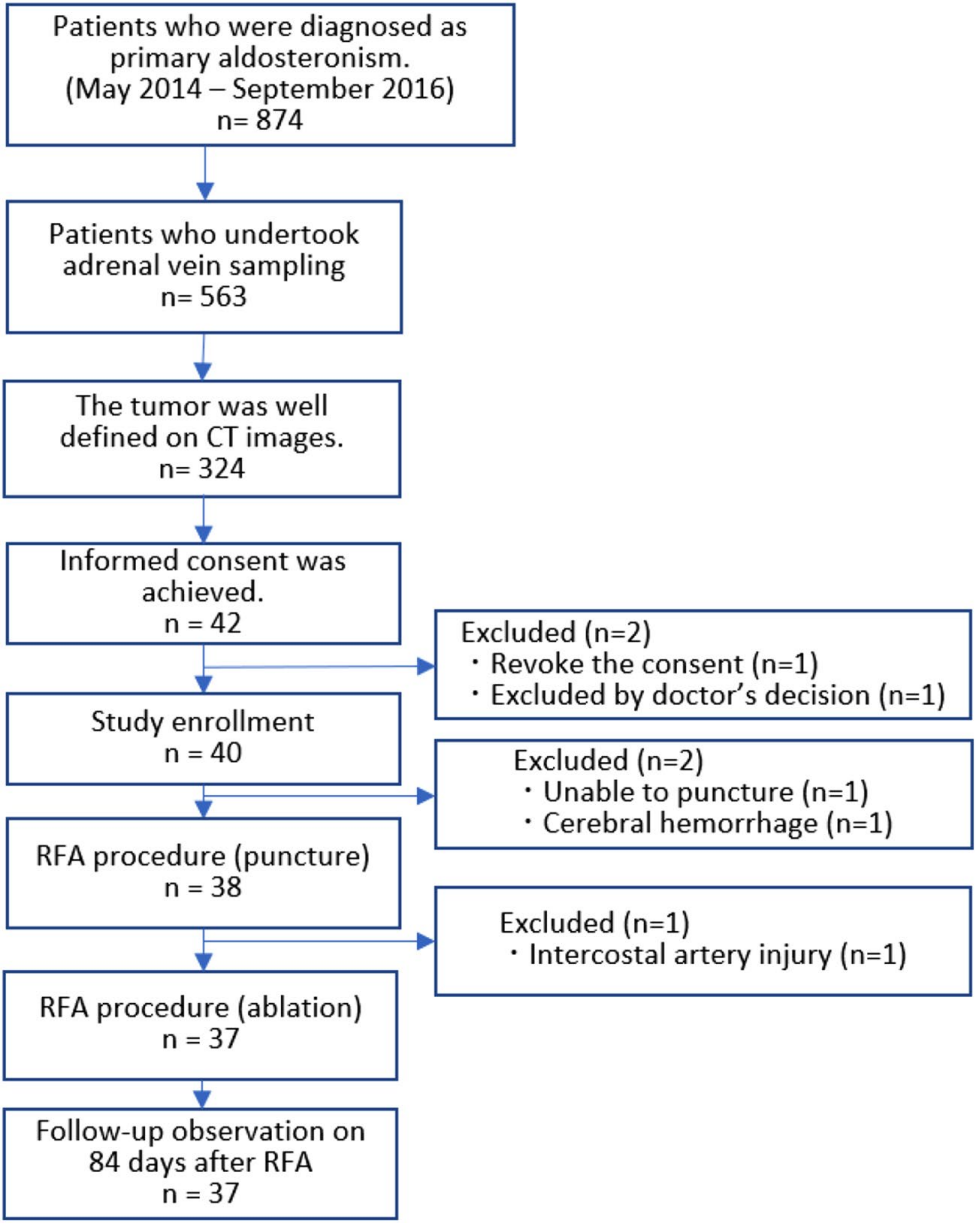
Study flow chart.

RFA was tried on the remaining 38 patients. An intercostal arterial injury occurred during the RFA needle puncture in one patient, which required arterial embolization; hence, the RFA procedure was aborted. Consequently, the RFA procedure was completed in 37 patients (Fig. 2). Therefore, 38 cases were analyzed for evaluation of the safety of the procedure, on the other hand, 37 cases were analyzed for evaluation of the feasibility and efficacy. Patient backgrounds and tumor characteristics are listed in Table 1.

**Table 1:** Patient backgrounds and tumor characteristics.

Patient characteristics	Number of patients	Mean values
Mean age (years)	37	50.4 ± 10.0
**Sex- male**	20	
Female	17	
Height (cm)	37	163.9 ± 8.7
Weight (kg)	37	66.0 ± 12.4
**Tumor size (mm)**	37	14.8 ± 3.8
Tumor size less than 10 mm	5	
Tumor size from 10 to 25 mm	32	
Plasma aldosterone concentration (ng/dL) on the treatment date	37	47.2 ± 31.5
Plasma aldosterone concentration (ng/dL) on day 84	37	10.83 ± 4.28
Plasma renin activity (ng/mL/hr) on the treatment date	37	0.50 ± 0.45
Plasma renin activity (ng/mL/hr) on day 84	37	0.95 ± 0.97
Urine potassium (mEq/L) on the treatment date	37	45.9 ± 30.8
Urine potassium (mEq/L) on day 84	37	50.7 ± 26.8
Plasma aldosterone concentration (μg/day) on the treatment day	37	30.7 ± 17.8
Plasma aldosterone concentration (μg/day) on day 3	37	3.6 ± 3.9
Systolic blood pressure on the treatment day (mmHg)	37	126.9 ± 14.9
Systolic blood pressure on day 84 (mmHg)	37	131.7 ± 13.5
Diastolic blood pressure on the treatment day (mmHg)	37	83.1 ± 11.0
Diastolic blood pressure on day 84 (mmHg)	37	86.2 ± 10.9

### RFA procedure

According to dynamic CT findings acquired 7 days after the 1st procedure, 36 out of 37 cases were judged as 100% ablation rate at individual institutions; the remaining case was judged as incomplete ablation rate that required 2nd session.

The mean number of ablation cycles was 3.1 ± 1.5 times (1–7 times). In nine out of 38 sessions (23.7%), the RFA procedure was completed in one ablation cycle. The ablation cycle was most commonly performed twice in 16 sessions (42.1%) with reposition of the electrodes. Output time per ablation cycle was 6.3 ± 1.9 min, the overall output time per session was 19.1 ± 9.4 min, and the total energy input was 17 ± 7.8 kJ. The ablation was temporarily interrupted by a pain in 11 sessions and the NIBP elevation in 8 sessions and an elevation of the impedance in 2 sessions. The total procedure time was 163.3 ± 51.6 min.

### Adverse events

Intraprocedural hypertension > 180 mmHg was observed during RFA in 16 of 38 sessions (42.1%). The highest values of systolic NIBP during the procedure was from 110 to 270 mmHg and they were measured while the tumor was ablated in 30 out of 38 sessions (78.9%). In other 8 sessions, they were observed before the local anesthesia. The difference between the highest and lowest systolic NIBP was 83.4 ± 36.0. The highest elevation of systolic NIBP during ablation was 171 mmHg at the ablation time.

As for the evaluation of the safety of the procedure of 38 cases who were tried to perform the RFA, other than intraprocedural hypertension, grade 3 adverse events were observed in 10 out of 38 cases (26.3%), grade 4 adverse events were observed in 3 out of 38 cases (7.9%), and Grade 5 adverse events were not observed. Grade 4 was defined as one case of apnea, one case of cholecystitis, and one case of thoracic bleeding, intercostal arterial injury, and hemorrhagic shock, which required transcatheter arterial embolization. Apnea and cholecystitis occurring 78 days after the RFA procedure were considered unrelated to the procedure. Thoracic bleeding with hemorrhagic shock due to intercostal arterial injury was judged as a major complication during the insertion of the RF applicator. Therefore, intercostal arterial injury was the only adverse event related to the RF procedure, seen in 2.6% patients (1/38).

### Third-party evaluation of the ablated tumor volume and clinical outcome

The third-party organization reviewed CT images acquired 7 days after the completion of each RFA session to evaluate ablated tumor volume independently. Out of 36 cases who underwent only one RFA sessions, ablated tumor volumes were judged as 100% in 34 cases, 99.6% in one case, and 94.8% in the remaining case. One case required 2nd RFA session; the ablated tumor volume was judged as 48.1% at the 1st procedure and 100% at the 2nd procedure. Therefore, the technical success rate of RFA was 94.6% (35/37).

**Figure 3:**
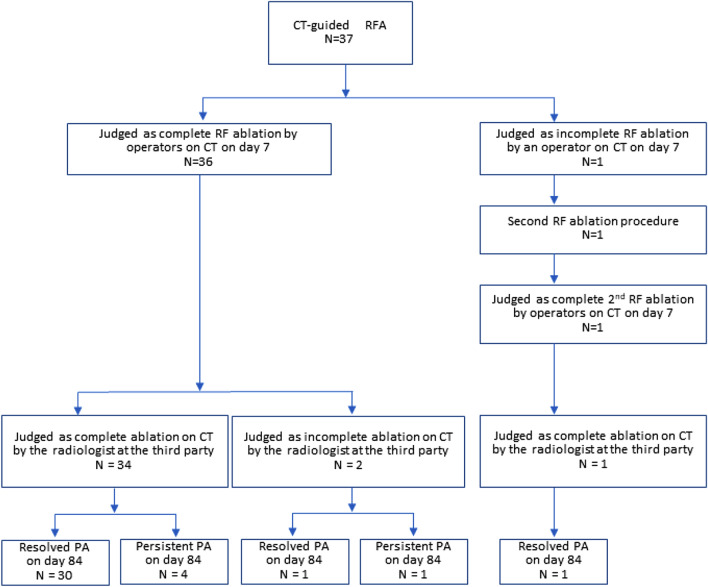
Flow charts results of RF procedure at 84 days.

Clinical outcomes according to the ablated tumor volumes are as follows: normalized hormone activity on day 84 was observed in 31 out of 35 cases with technical success (100% tumor volume) and in one out of two cases with incomplete ablation (< 100% ablated tumor volume) (Fig. 3).

**Figure 4:**
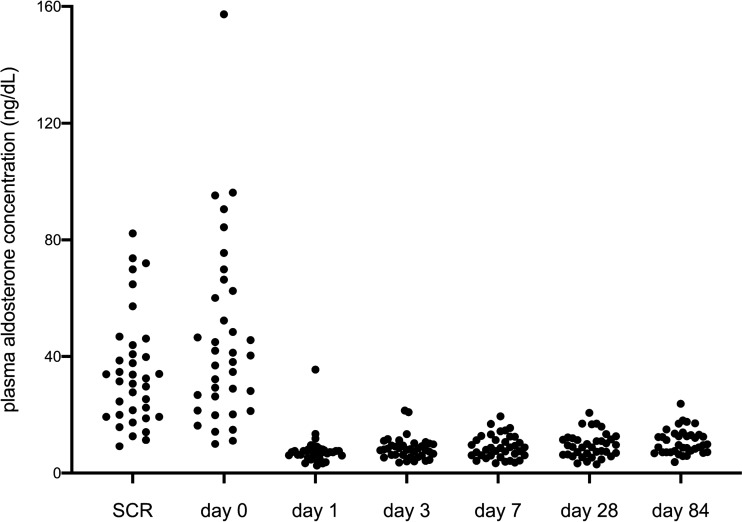
The results of transition of aldosterone concentration from day0 to 84 day after RF procedure. SCR: Day of screening for primary aldosteronism.

The results of the aldosterone concentration transition from day 0 to 84 days after the RF procedure are shown in Fig. 4. Plasma aldosterone concentration or ARR was normalized in 97.2% (85.8–99.5%: 95% confidence interval) patients on day 1, and was normalized in 86.5% (72.0–94.1%: 95% confidence interval) on day 84. A total of 32 out of 37 cases (86.5%) reached normalized excretion of aldosterone on day 84, including one case with 99.6% ablated volume.

The number and types of antihypertensive drugs showed a significant decrease from 1.6 ± 1.3 on day 0 to 0.5 ± 0.7 on 84 days after the procedure (*p* < 0.001). The number and types of antihypertensive drugs required was 0 in 33 cases on day 1 and in 22 cases on day 84. The outcomes of CT-guided percutaneous RFA are summarized in Table 2.

**Table 2:** Outcomes of CT-guided percutaneous radiofrequency ablation.

Outcomes	
Mortality	0%
Intraprocedural hypertension over 180 mmHg	43.2%
**Adverse events**	
Grade 3	26.3%
Grade 4	2.6%
Grade 5	0%
**Treatment success rate**	
Percentage of complete tumor ablation on CT scan, (%)	94.6%
Percentage of aldosterone normalization rate at 84 days, (%)	86.5%
**The number of types of antihypertensive drugs**	
Day 0	1.6 ± 1.3
Day 84	0.5 ± 0.7

*CT* Computed tomography.

## Discussion

This multicenter prospective study shows that percutaneous CT-guided RFA for APA using bipolar radiofrequency system achieved normalization of plasma aldosterone concentration or aldosterone-renin ratio of 86.5% (72.0–94.1: 95% confidence interval) on day 84, which was defined as the primary endpoint. Based on a previous study, unilateral adrenalectomy for APA resulted in eradication of hypokalemia and resolution or significant improvement in hypertension in 80% patients at long-term follow-up^20^. Therefore, our study indicated that clinical outcome by bipolar RFA could be achieved comparable with surgical adrenalectomy. Our results also indicate that percutaneous RFA for APAs would significantly reduce the number of antihypertensive drugs and aldosterone antagonists prescribed in patients with primary hyperaldosteronism.

There have been multiple reports of RFA for adrenal adenomas using monopolar RF devices^10–14,24–26^. They treated APA of the size from 4 to 34 mm. Mortality and major morbidity was not observed in any reports, and minor morbidity ranged 15–24%. In this study, major morbidity was observed in one intercostal arterial injury case, minor morbidity was 26%. Intercostal arterial injury could happen when inserting the needle through the intercostal area, especially in CT guided procedure, because arteries cannot well be visualized in non-contrast enhanced CT images. Minor morbidity rate in this study was comparable in previous reports. More recently, Liu et al*.* reported the use of a monopolar RF system for APA in 36 cases with a mean follow-up duration of 6.2 years in a single facility^24^. The long-term recurrence rate was 0%. Therefore, CT-guided RF ablation was considered as an effective treatment for APA with high sustainable long-term treatment success. However there have been reported only single center study using monopolar devices. This study was unique in terms of its multicenter prospective study design and the use of a bipolar system for RFA of APA.

The advantage of the bipolar RFA system over the monopolar system has been reported in the treatment of hepatocellular carcinoma. Chang et al*.* showed that the bipolar RFA systems required a smaller number of ablation cycles and shorter ablation time than the monopolar RFA systems^27^. However, in this study, RFA sessions were needed more than once per ablation session in 76.3%. A potential reason for requiring multiple sessions of RFA in the adrenal gland tumor was that the adrenal gland has one of the largest blood supply rates per gram of the organ^28^. Furthermore, RFA sessions were added up to 7 until tumor enhancement disappeared on intraprocedural dynamic CT. This strict treatment policy might be one of the reasons why the overall output time per session was slightly longer (19.1 min) than that in other reports (10–12 min)^24–26^.

Regarding complications, intercostal artery injury occurred in one case in this study (2.6%). In a previous study, the rate of major complications caused by RFA, including intercostal artery injury and abscess formation, was reported to range from 0 to 11.5%^12,24,27,29^. Therefore, this procedure should be performed by a skilled interventional team that has extensive experience with CT-guided procedures in a facility with a CT fluoroscopy and angiography system that helps in case of possible emergent vascular embolization of unexpected bleeding.

From our results, intraprocedural hypertension > 180 mmHg was observed in 43% of cases, whereas a previous study reported that it was observed in only 8–20% of the cases^12,25^. The reason for this discrepancy is unknown; however, in this study, systolic NIBP was measured continuously from the beginning to the end of the ablation cycle. This might have led to the detection of intraprocedural hypertension occurring for a short time. Another possibility in this study is that many APAs were located close to the adrenal medulla, or a wider range was ablated by the bipolar RFA system, which caused ablation of the adrenal medulla. However, these probabilities were not examined in this study and should be discussed in future. As for using the usage of premedication, alpha blockers are commonly used as antihypertensive drugs, so administering them in advance is worthy of consideration. On the other hand, since catecholamine secretion during ablation occurs instantaneously, intravenous administration of fast-acting drugs is desirable. It is difficult to sufficiently suppress a hypertensive crisis with a regular dose of alpha blocker, because of a large amount of catecholamine release during RFA. Therefore, as in the present protocol, intravenous administration of the antihypertensive drug during the procedure would be a more appropriate method to the hypertensive crisis caused by RFA.

Our study had limitations. First, the follow-up period was shorter than that in previous studies. Since this study was prospective study and was already censored, the follow-up period cannot be extended, but studies on long term outcome are planned. Similar to the study by Liu et al., the effect of RFA on APA is expected to be long-lasting^24^. However, as all patients in our study were on clinical follow-up, long-term results of our cohorts will be presented in future. Second, there has been no comparison between RFA and surgical approach in terms of clinical outcome and complications. In the current situation, surgical adrenalectomy is the gold standard. However, a randomized study would be warranted to investigate whether less invasive RFA procedures may be considered as the first line option for the treatment, by replacing the surgery in the future. Third, histological proof of the adrenal tumor was not obtained in any case. Malignant adrenal tumors were not completely ruled out by histological evaluation. However, aldosterone-producing adrenocortical carcinomas are extremely rare and the tumor size to be ablated is so small that malignancy is clinically negligible. Forth, a trans-pulmonary puncture was not included in this study. Once the feasibility of RFA is revealed, a further study should be warranted to evaluate efficacy and safety including trans-pulmonary puncture.

In conclusion, percutaneous CT-guided RFA for APA using a bipolar radiofrequency system was safe and feasible, achieving clinical success rate of 86.5% on day 84. Percutaneous RFA could be a less invasive therapeutic option for treating APAs.

## Data Availability

The datasets generated during and/or analyzed during the current study are available from the corresponding author on reasonable request.

## Abbreviations

RFA: Radiofrequency ablation
APA: Aldosterone-producing adenoma
PA: Primary hyperaldosteronism
AVS: Adrenal vein sampling
NIBP: Non-invasive blood pressure
ARR: Aldosterone-renin ratio
